# Demographic and Substance Use Factors Associated with Non-Violent Alcohol-Related Injuries among Patrons of Australian Night-Time Entertainment Districts

**DOI:** 10.3390/ijerph14010075

**Published:** 2017-01-12

**Authors:** Kerri Coomber, Richelle Mayshak, Shannon Hyder, Nicolas Droste, Ashlee Curtis, Amy Pennay, William Gilmore, Tina Lam, Tanya Chikritzhs, Peter G. Miller

**Affiliations:** 1 School of Psychology, Faculty of Health, Deakin University, Geelong VIC 3220, Australia; richelle.mayshak@deakin.edu.au (R.M.); shannon.hyder@deakin.edu.au (S.H.); nic.droste@deakin.edu.au (N.D.); ashlee.curtis@deakin.edu.au (A.C.); A.Pennay@latrobe.edu.au (A.P.); petermiller.mail@gmail.com (P.G.M.); 2 Centre for Alcohol Policy Research, Department of Psychology and Public Health, La Trobe University, Melbourne VIC 3000, Australia; 3 National Drug Research Institute, Curtin University, Perth WA 6845, Australia; william.gilmore@curtin.edu.au (W.G.); tina.lam@curtin.edu.au (T.L.); t.n.chikritzhs@curtin.edu.au (T.C.)

**Keywords:** alcohol, intoxication, injury, licensed venues, patron interviews

## Abstract

This study examined the relationship between patron demographics, substance use, and experience of recent alcohol-related accidents and injuries that were not due to interpersonal violence in night-time entertainment districts. Cross-sectional interviews (*n* = 4016) were conducted around licensed venues in entertainment districts of five Australian cities. Demographic factors associated with non-violent alcohol-related injuries were examined, including gender, age, and occupation. The association between substance use on the night of interview; blood alcohol concentration (BAC), pre-drinking, energy drink consumption, and illicit drug use; and experience of injury was also explored. Thirteen percent of participants reported an alcohol-related injury within the past three months. Respondents aged younger than 25 years were significantly more likely to report an alcohol-related injury. Further, a significant occupation effect was found indicating the rate of alcohol-related injury was lower in managers/professionals compared to non-office workers. The likelihood of prior alcohol-related injury significantly increased with BAC, and self-reported pre-drinking, energy drink, or illicit drug consumption on the night of interview. These findings provide an indication of the demographic and substance use-related associations with alcohol-related injuries and, therefore, potential avenues of population-level policy intervention. Policy responses to alcohol-related harm must also account for an assessment and costing of non-violent injuries.

## 1. Introduction

Alcohol-related injuries account for more than one third of the burden of disease associated with alcohol consumption worldwide, with injury causes including violence against self or others, road traffic accidents, burns, poisoning, and falls [1]. A meta-analysis of 150 studies indicated that a blood alcohol concentration (BAC) of ≥0.10 g/100 mL was related to 31.5% of homicide related deaths, 31.0% of non-traffic unintentional deaths, and 22.7% of suicide cases [2]. Alcohol-related injuries are commonly evidenced by emergency department presentations, with young males typically accounting for the majority of alcohol-related injury emergency department presentations [3,4,5]. 

Injuries occurring in and around night-time entertainment districts typically occur Friday, Saturday, and Sunday nights between midnight and 3 a.m. [6,7], and have been associated with excess alcohol consumption within licensed premises and in public spaces [8]. Particularly within licensed premises, a BAC ≥ 0.08 g/100 mL is associated with an increased risk of injuries due to interpersonal violence [5]. A recent US study also found that among bar patrons for each unit increase in breath alcohol concentration there was over twice the risk of reporting an alcohol-related injury in the past 12 months [9]. Further, one meta-analysis indicates that the odds of presenting at an emergency department with an injury increase by 1.30 for every 10 g increase in alcohol consumption [10]. Therefore, patterns of risky drinking in night-time entertainment districts is a particular cause for concern. 

Pre-drinking, or consuming alcohol before attending licensed premises, has been found to be associated with heavy drinking for the remainder of the evening for both men and women, increased drink-driving, illicit drug use, and aggressive encounters between groups of young males [11]. However, little research has been conducted examining pre-drinking and its relationship with risk of non-violent injury, with much of the research into pre-drinking and intoxication-related injuries conducted outside of Australia, e.g., [12]. Therefore, the current study aims to address this gap in the literature.

While only a small proportion (7%) of Australian patrons in night-time entertainment districts report using illicit drugs on a given night, the use of such drugs has been found to almost double the risk of involvement in a violent incident for young males [13,14,15,16]. In addition, the combined consumption of alcohol mixed with energy drinks (AmED) has been shown to lead to heavy drinking, risky behaviours, and an increased prevalence of negative outcomes such as violent injuries and accidents [17,18]. Energy drinks increase stimulation (wakefulness, energy, alertness) and are typically used to facilitate users to stay out late [17]. Consuming AmED may mask the feelings of intoxication and lead to greater consumption of alcohol over a longer period of time [19], and when combined with the reduced cognitive and verbal capacity associated with alcohol intoxication, the likelihood of conflict is substantially increased [14,20]. However, there is a lack of research examining the role of consuming other substances, such as illicit drugs and energy drinks, on the likelihood of experiencing non-violent alcohol-related injury. 

Alcohol-related injuries cause significant social and economic harm, and place substantial strain on emergency departments. However, the majority of research within night-time entertainment districts has typically focussed on injury due to interpersonal violence [6,21], with a lack of knowledge regarding alcohol-related injuries that are not related to interpersonal violence. The current brief study uses patron interview data from a large investigation of night-time entertainment districts (the Patron Offending and Intoxication in Night Time Entertainment Districts (POINTED) study [19]) to examine the relationship between patron demographic and substance use on the night of the interview, and experience of non-violent alcohol-related injuries in the past three months.

## 2. Materials and Methods

### 2.1. Setting

Five Australian cities were chosen for the current study. These locations were selected to enable comparison between large metropolitan cities (Sydney and Melbourne), with smaller regional cities within the same jurisdictions (Wollongong and Geelong). Perth was also selected as it is a unique smaller metropolitan city on the opposite coast of Australia. Each city has busy night-time entertainment districts with late trading venues (i.e., venues open until at least 3 a.m.).

### 2.2. Procedure

Ethics approval (EC00213; 2011-095) was obtained from all participating institutions (*n* = 6). Full details of the POINTED methodology can be found in a published study protocol [19]. Interview sessions were undertaken between December 2011 and July 2012 in all cities on a fortnightly basis on Friday and Saturday nights, and also less frequently on Wednesday and Thursday nights. Interviews were conducted from 6 p.m. to 3 a.m. (95% of interviews were conducted between 10 p.m. and 2 a.m.) with patrons in busy thoroughfares of entertainment districts in each city (*n* = 4216). The interview response rate was 93% [22]. In order to obtain a systematic random sample, every third person was invited to take part. Respondents were given a business card with study details and contact details of the investigators and ethics committee. Interviews were completed using a survey application (Tap Forms™, Tap Zapp Software, Calgary, AB, Canada) on a mobile device. Patrons were also asked to provide an estimate of blood alcohol concentration (BAC) measure using a calibrated breathalyser.

### 2.3. Measures

Demographics: All respondents were asked their gender and age (categorised into 18–20 years, 21–25 years, and >25 years). Respondents were also asked their current occupation. This was then categorised using the Australian and New Zealand Standard Classification of Occupations (ANZSCO) [23]. For analysis, we created six groups: “managers/professional”; “non-office worker” (including technicians and trades workers, machinery operators and drivers, and labourers); “community and personal service worker”; “sales/clerical/administrative worker”; “student” and, “other” (where the job provided was not in the ANZSCO list, not valid, or the respondent was retired).

Substance use measures on the night of interview: Respondents were asked if they consumed alcohol prior to going out to a licensed venue (e.g., in a private home or other private setting; pre-drinking), whether they had consumed any energy drinks during the night (including AmED), and whether they had taken any other drugs prior to the interview, including illicit drugs or prescription drugs not prescribed to them (including ecstasy, cocaine, methamphetamines, pharmaceutical stimulants, ketamine, Lysergic acid diethylamide (LSD), Gamma-Hydroxybutyric acid (GHB), benzodiazepines, heroin, cannabis, mephedrone, or other illicit substance). While it would be valuable to examine the impact of different drug types on experience of alcohol-related injury, the number of participants responding “yes” to individual drug types was quite low (for the majority of individual substances there were less than 1% responding “yes”, with ecstasy and cannabis having the highest proportions at 3% each). Therefore, the impact of specific drug types were not able to be investigated and the overall drug measure has been used.

Outcome measure: Respondents were asked: “Have you had any alcohol-related injuries or accidents in the past three months?” The outcome variable was coded as “yes” versus “no”.

### 2.4. Analysis

Analyses were conducted using Stata 14.0 (StataCorp, College Station, TX, USA). Prior to analysis multicollinearity between the independent variables was tested; the variance inflation factors were low (mean = 1.10; range = 1.03–1.15), indicating multicollinearity was not present in the data. A Box-Tidwell test was then conducted to determine if BAC could be modelled as a continuous numerical variable; this was non-significant (*p* = 0.607). Therefore, BAC was modelled as a continuous measure with odds ratios scaled to represent an increased risk for a 0.01 increase in BAC. Two multivariate logistic regression models were conducted. The first model examined the associations between the demographic variables (i.e., gender, age, and occupation) and outcome variable. The second examined the association between current night out substance use and alcohol-related injuries, while controlling for demographic variables. Each model controlled for clustering by city in order to account for potential intra-group (i.e., city) correlations among patron responses, and time of interview.

## 3. Results 

### 3.1. Demographics

The majority of participants were male, with approximately one-third aged 18–20 years (Table 1). More than half of respondents reported pre-drinking, with the average BAC measuring 0.056 g/100 mL (standard deviation (SD) = 0.053). This average BAC is above the legal driving limit in Australia of 0.050 g/100 mL. Most (81%) had not consumed energy drinks on the night, and 9% of respondents had taken illicit drugs prior to the interview. Thirteen percent of respondents reported being involved in an alcohol-related injury during the past three months.

### 3.2. Demographic Factors Associated with Non-Violent Alcohol-Related Injuries in the Past Three Months

A multivariate logistic regression model indicated no difference in the proportion of males and females experiencing an alcohol-related injury (Table 2). However, respondents younger than 25 years were significantly more likely to be involved in an alcohol-related injury compared to those age over 25 years (Table 2). Further, compared to managers/professionals, non-office workers, community and personal service workers, and sales/clerical/administration workers were significantly more likely to report involvement in alcohol-related injuries. The model was then re-run using non-office workers as the reference group; compared to non-office workers, those categorised within the “other” category were significantly less likely experience an alcohol-related injury in the past three months (odds ratio (OR) = 0.76, 95% confidence interval (CI) = 0.58–0.98, *p* = 0.036). There was no difference between non-office workers and community and personal service workers, or sales/clerical/administration workers, or students on rates of injury.

### 3.3. Current Night Substance Use Factors Associated with Non-Violent Alcohol-Related Injuries in the Past Three Months

A second multivariate model indicated that for each 0.01 g/100 mL increase in BAC, there was a 2.0% increased likelihood of reporting a non-violent injury due to alcohol intoxication (Table 3). In addition, respondents who had engaged in pre-drinking, consumed energy drinks during the night, or consumed illicit drugs had significantly higher odds of involvement in an alcohol-related injury.

## 4. Discussion

Approximately one in seven interviewees in Australian night-time entertainment districts reported having experienced a non-violent alcohol-related injury in the past three months. This study found that respondent age and occupation were significantly associated with involvement in an alcohol-related injury. Further, BAC, pre-drinking, drug use, and energy drink use on the night of interview were also associated with a higher odds of alcohol-related injury. These findings provide a unique perspective on harms experienced by people attending night-time entertainment districts, providing useful information beyond hospital and ambulance records. It also serves as a useful reminder that alcohol-related harms from incidents such as simply falling over and injuring oneself are at least as common as those arising from violence [22].

As anticipated [3,4,5], patrons younger than 25 years were had significantly higher odds of being involved in an alcohol-related injury. This is likely to be due, in part, to the higher rates of risky drinking among this age group [24]. This is also consistent with prior research finding that younger patrons are more likely to be involved in aggressive incidents in and around licensed venues [25,26,27]. A novel finding of the current study was that respondents employed in occupations categorised as non-office, community and personal service, and sales/clerical/administration were more likely to report experiences of alcohol-related injury in the past three months. This pattern of findings is consistent with that found for alcohol-related aggression [25,28] and may be partially driven by socio-economic disadvantage [25]. Research indicates that people from low socio-economic backgrounds are more likely to consume alcohol at high-risk levels [29,30], therefore placing them at an increased risk of alcohol-related injury. Further research is required to explore the effects of occupation and socio-economic status on alcohol-related injuries. 

Consistent with previous research [5,9,31], the current study showed that higher BAC on the night of interview was associated with alcohol-related injuries in the past three months. Also consistent with the literature [13,17,19], pre-drinking, consumption of energy drinks, and illicit drug taking on the night of the interview were associated with an increased risk of alcohol-related injuries. These practices are all associated with increased intoxication e.g., [11,17], which might explain their association with alcohol-related injuries. While our findings relate current night substance use to harms in the past three months, they do indicate that a focus on limiting the availability of alcohol, through avenues such as restricted trading hours and increased price, are likely to be effective in reducing alcohol-related injuries [32]. 

## 5. Limitations

It should be noted that this study used measurements of behaviour on the night of the interview to form associations with incidents of injury occurring in the past three months. This approach was taken as the prevalence of reported incidents of injury on a given night is likely to be low. This is due to interviewing people during the course of the night and not at the end of their night out, and also as if the injury was severe enough to warrant ambulance or hospital attendance these people are unlikely to return to the entertainment district after the incident and, thus, available to be interviewed. While such a method does not allow for conclusions regarding direct associations between substance use and injury on a specific night, it does allow associations between substance use and prior experience of injury to be explored. Therefore, this approach assumes behaviour on the night of the interview represents usual substance use behaviour for respondents when they are in night-time entertainment districts, which is a method often used in studies of this kind [13,33,34]. It must also be noted that experience of prior alcohol-related injury may have resulted in some respondents reducing their current alcohol use [35]. 

While patron interviews, or “portal studies” [36] have a number of advantages over traditional telephone or household surveys, including reduced recall bias, more objective data, the ability to make environmental observations and especially the ability to link specific characteristics of the night (such as BAC) to specific outcomes (such as injuries), there are some limitations. For instance, such surveys do not necessarily represent all people who attend licensed venues, although our response rate of over 90% is promising. Further, as the interviews for the current study were conducted within a comparatively public environment some participants may not have responded truthfully. Additionally, the nature and severity of the alcohol-related injuries incurred was not assessed. What constitutes an injury may differ between individuals or groups. Further, we have made the assumption that participants are reporting on non-violent incidents; it may be that in some cases participants are referring to incidents of self-harm. While the collection of real-time experience of injury/accident would be ideal, the resources required to conduct such a study are prohibitive, although studies collecting “last drinks” information about sources of alcohol-related injuries are proving useful and deserve replication [37,38]. The location of where the alcohol-related injury occurred was also not collected. While the current paper focusses on the context of night-time entertainment districts, it may be that an alcohol-related injury occurred at the respondent’s home.

## 6. Conclusions

Experiences of non-violent alcohol-related injury is common for night-time entertainment district attendees, and are often an overlooked form of social harm. While age and occupation were found to be associated with alcohol-related injuries, pre-drinking and substance use, along with increasing BAC are strong contextual contributors amenable to intervention. As past risky substance use is a significant predictor of future substance use [39,40,41], and therefore the likelihood of harms, these findings provide an indication of the demographic and substance use-related associations with alcohol-related injuries and, therefore, the types of population-level policy interventions that have the potential to reduce such harms. These findings provide further support for the use of interventions that regulate the price and availability of alcohol. Policy responses to alcohol-related harm associated must also include assessments and costings of injuries, as well as violence and aggression.

## Figures and Tables

**Table 1:** Patron characteristics.

Variable	*n*	%
Gender		
Male	2436	58
Female	1739	42
Age		
18–20 years	1222	29
21–25 years	1583	38
>25 years	1353	33
Occupation		
Managers/professionals	1083	26
Non-office workers	755	18
Community and personal service worker	381	9
Sales/clerical/administrative worker	546	13
Student	1116	27
Other or unemployed	245	6
Consumed alcohol prior to going out to licensed venues	2325	55
Consumed energy drinks during the night	822	20
Taken other drugs during the night	390	9
Outcome: Involved in an accident or injury in past three months	517	13

Note. *n* range = 3875 to 4216 due to missing data.

**Table 2:** Multivariate logistic regression models examining demographic factors associated with involvement in a non-violent alcohol-related injury in the past three months.

Variable	OR (95% CI)	*p*-Value	% Reported Alcohol-Related Injury
Gender			
Male	1.00		13
Female	1.10 (0.97, 1.24)	0.121	14
Age	Wald χ^2^ *p* < 0.001		
>25 years	1.00		8
21–25 years	2.43 (1.86, 3.16)	<0.001	14
18–20 years	1.84 (1.56, 2.17)	<0.001	18
Occupation	Wald χ^2^ *p* < 0.001		
Managers/professionals	1.00		11
Non-office workers	1.46 (1.03, 2.09)	0.032	16
Community and personal service worker	1.62 (1.22, 2.15)	0.001	17
Sales/clerical/administrative worker	1.42 (1.08, 1.87)	0.012	15
Student	1.04 (0.80, 1.34)	0.788	12
Other or unemployed	1.11 (0.75, 1.64)	0.593	13

Note. Models also controls for time of interview and clustering by city; *n* = 3756. Cox-Snell *R*^2^ = 0.017; Nagelkerke *R*^2^ = 0.032. OR = odds ratio; 95% CI = 95% confidence interval.

**Table 3:** Multivariate logistic regression models examining current night out substance use associated with involvement in a non-violent alcohol-related injury in the past three months.

Variable	OR (95% CI)	*p*-Value	% Reported Alcohol-Related Injury
Blood alcohol concentration (BAC) ^a^	1.02 (1.01, 1.04)	<0.001	--
Consumed alcohol prior to going out to licensed venues			
No	1.00		12
Yes	1.25 (1.11, 1.42)	<0.001	14
Consumed energy drinks during the night			
No	1.00		12
Yes	1.59 (1.22, 2.01)	0.001	18
Taken other drugs during the night			
No	1.00		13
Yes	1.35 (1.22, 1.48)	<0.001	17

^a^ The odds ratio for blood alcohol concentration (BAC) was scaled such that it represents the odds of involvement in an incident for each 0.01 increase in BAC. This was determined through using the following calculation: model OR^0.01. -- Percentage not reported due to BAC modelled as a continuous variable. Note. Model also controls for gender, age, occupation, time of interview, and clustering by city; *n* = 3678. Cox-Snell *R*^2^ = 0.028; Nagelkerke *R*^2^ = 0.051. OR = odds ratio; 95% CI = 95% confidence interval.

## References

[1] Cherpitel C.J. (2009). Alcohol and Injuries: Emergency Department Studies in an International Perspective.

[2] Smith G.S., Branas C.C., Miller T.R. (1999). Fatal nontraffic injuries involving alcohol: A metaanalysis. Ann. Emerg. Med..

[3] Cherpitel C.J. (1993). Alcohol and violence-related injuries: An emergency room study. Addiction.

[4] Cherpitel C.J. (2007). Alcohol and injuries: A review of international emergency room studies since 1995. Drug Alcohol Rev..

[5] Macdonald S., Cherpitel C.J., Borges G., DeSouza A., Giesbrecht N., Stockwell T. (2005). The criteria for causation of alcohol in violent injuries based on emergency room data from six countries. Addict. Behav..

[6] Chikritzhs T., Stockwell T. (2002). The impact of later trading hours for Australian public houses (hotels) on levels of violence. J. Stud. Alcohol.

[7] Ireland C., Thommeny J. (1993). The crime cocktail: Licensed premises, alcohol and street offences. Drug Alcohol Rev..

[8] Graham K., Homel R. (2008). Raising the Bar: Preventing Violence in and around Bars, Pubs and Clubs.

[9] Martin R.J., Brechbiel K., Chaney B.H., Cremeens-Matthews J., Vail-Smith K. (2016). Alcohol-related injuries, hazardous drinking, and BrAC levels among a sample of bar patrons. Am. J. Addict..

[10] Taylor B., Irving H.M., Kanteres F., Room R., Borges G., Cherpitel C., Greenfield T., Rehm J. (2010). The more you drink, the harder you fall: A systematic review and meta-analysis of how acute alcohol consumption and injury or collision risk increase together. Drug Alcohol Depend..

[11] Wells S., Graham K., Purcell J. (2009). Policy implications of the widespread practice of “pre-drinking” or “pre-gaming” before going to public drinking establishments—Are current prevention strategies backfiring?. Addiction.

[12] Labhart F., Graham K., Wells S., Kuntsche E. (2013). Drinking before going to licensed premises: An event-level analysis of predrinking, alcohol consumption, and adverse outcomes. Alcohol. Clin. Exp. Res..

[13] Schnitzer S., Bellis M.A., Anderson Z., Hughes K., Calafat A., Juan M., Kokkevi A. (2010). Nightlife violence: A gender-specific view on risk factors for violence in nightlife settings: A cross-sectional study in nine European countries. J. Interpers. Violence.

[14] Miller P., Droste N., Martino F., Palmer D., Tindall J., Gillham K., Wiggers J. (2014). Illicit drug use and experience of harm in the night-time economy. J. Subst. Use.

[15] Miller P., Tindall J., Sonderlund A., Groombridge D., Lecathelinais C., Gillham K., MacFarlane E., de Groot F., Droste N., Sawyer A. (2012). Dealing with Alcohol-Related Harm and the Night-Time Economy (DANTE): Final Report.

[16] Miller P., Curtis A., Jenkinson R., Droste N., Bowe S.J., Pennay A. (2015). Drug use in Australian nightlife settings: Estimation of prevalence and validity of self-report. Addiction.

[17] Brache K. (2014). Alcohol and Energy Drinks: Motivations, Drinking Behaviours and Associated Risks.

[18] Miller K.E., Quigley B.M., Eliseo-Arras R.K., Ball N.J. (2016). Alcohol mixed with energy drink use as an event-level predictor of physical and verbal aggression in bar conflicts. Alcohol. Clin. Exp. Res..

[19] Miller P., Pennay A., Jenkinson R., Droste N., Chikritzhs T., Tomsen S., Wadds P., Jones S., Palmer D., Barrie L. (2013). Patron offending and intoxication in night time entertainment districts (POINTED): A study protocol. Int. J. Alcohol Drug Res..

[20] Pennay A., Miller P., Busija L., Jenkinson R., Droste N., Quinn B., Jones S.C., Lubman D.I. (2015). “Wide-awake drunkenness”? Investigating the association between alcohol intoxication and stimulant use in the night-time economy. Addiction.

[21] Graham K., Bernards S., Osgood D.W., Wells S. (2006). Bad nights or bad bars? Multi-level analysis of environmental predictors of aggression in late-night large-capacity bars and clubs. Addiction.

[22] Miller P., Pennay A., Droste N., Jenkinson R., Chikritzhs T., Tomsen S., Wadds P., Jones S., Palmer D., Barrie L. (2013). Patron Offending and Intoxication in Night Time Entertainment Districts (POINTED): Final Report.

[23] Australian Bureau of Statistics (2013). Cat No. 1220.0 ANZSCO: Australian and New Zealand Standard Classification of Occupation.

[24] Australian Institute of Health and Welfare (2014). National Drug Strategy Household Survey Detailed Report 2013, Drug Statistics.

[25] Zinkiewicz L., Curtis A., Meurer H., Miller P. (2015). Demographic risk factors for alcohol-related aggression in and around licensed venues. Alcohol Alcohol..

[26] Hughes K., Anderson Z., Morleo M., Bellis M.A. (2008). Alcohol, nightlife and violence: The relative contributions of drinking before and during nights out to negative health and criminal justice outcomes. Addiction.

[27] Hughes K., Bellis M.A., Calafat A., Juan M., Schnitzer S., Anderson Z. (2008). Predictors of violence in young tourists: A comparative study of British, German and Spanish holidaymakers. Eur. J. Public Health.

[28] Du Plessis K., Corney T., Burnside L. (2013). Harmful drinking and experiences of alcohol-related violence in Australian male construction industry apprentices. Am. J. Men's Health.

[29] Pollack C.E., Cubbin C., Ahn D., Winkleby M. (2005). Neighbourhood deprivation and alcohol consumption: Does the availability of alcohol play a role?. Int. J. Epidemiol..

[30] Jonas H., Dietze P., Rumbold G., Hanlin K. (1999). Associations between alcohol related hospital admissions and alcohol consumption in Victoria: Influence of socio-demographic factors. Aust. N. Z. J. Public Health.

[31] Galbraith S., Murray W., Patel A., Knill-Jones R. (1976). The relationship between alcohol and head injury and its effect on the conscious level. Br. J. Surg..

[32] Babor T. (2010). Alcohol: No Ordinary Commodity: Research and Public Policy.

[33] Miller P., Droste N., Groot F., Palmer D., Tindall J., Busija L., Hyder S., Gilham K., Wiggers J. (2015). Correlates and motives of pre-drinking with intoxication and harm around licensed venues in two cities. Drug Alcohol Rev..

[34] Curtis A., Coomber K., Hyder S., Droste N., Pennay A., Jenkinson R., Mayshak R., Miller P.G. (2016). Prevalence and correlates of drink driving within patrons of Australian night-time entertainment precincts. Accid. Anal. Prev..

[35] Longabaugh R., Minugh P.A., Nirenberg T.D., Clifford P.R., Becker B., Woolard R. (1995). Injury as a motivator to reduce drinking. Acad. Emerg. Med..

[36] Voas R.B., Furr-Holden D., Lauer E., Bright K., Johnson M.B., Miller B. (2006). Portal surveys of time-out drinking locations a tool for studying binge drinking and AOD use. Eval. Rev..

[37] Droste N., Miller P., Baker T. (2014). Review article: Emergency department data sharing to reduce alcohol-related violence: A systematic review of the feasibility and effectiveness of community-level interventions. Emerg. Med. Australas..

[38] Miller P., Droste N., Baker T., Gervis C. (2015). Last drinks: A study of rural emergency department data collection to identify and target community alcohol-related violence. Emerg. Med. Australas..

[39] Danner U.N., Aarts H., de Vries N.K. (2008). Habit vs. intention in the prediction of future behaviour: The role of frequency, context stability and mental accessibility of past behaviour. Br. J. Soc. Psychol..

[40] Norman P., Conner M. (2006). The theory of planned behaviour and binge drinking: Assessing the moderating role of past behaviour within the theory of planned behaviour. Br. J. Health Psychol..

[41] Ouellette J.A., Wood W. (1998). Habit and intention in everyday life: The multiple processes by which past behavior predicts future behavior. Psychol. Bull..

